# Primer fabrication using polymerase mediated oligonucleotide synthesis

**DOI:** 10.1186/1471-2164-10-344

**Published:** 2009-07-31

**Authors:** Murray J Cairns, Torsten Thomas, Carolina E Beltran, Daniel Tillett

**Affiliations:** 1 Schizophrenia Research Institute, Sydney, NSW 2006, Australia; 2 School of Biomedical Sciences, The University of Newcastle, NSW 2308, Australia; 3 Centre for Marine Bio-Innovation, School of Biotechnology & Biomolecular Sciences, The University of New South Wales, Sydney, NSW 2052, Australia; 4 Nucleics Pty Ltd, 12 Mill St, Bendigo, VIC 3550, Australia; 5 School of Pharmacy & Applied Science, La Trobe University, Bendigo, Victoria 3552, Australia

## Abstract

**Background:**

Custom solid phase oligonucleotide synthesis is an important foundation supporting nearly every aspect of current genomics. In spite of the demand for oligonucleotide primers, their synthesis remains relatively expensive, time consuming and in many circumstances a wasteful process. In this methodology, described as polymerase mediated oligonucleotide synthesis (PMOS), a DNA polymerase is used to increase the hybridization affinity of one oligonucleotide by using another as a template for DNA synthesis. This self-assembly process provides an opportunity to instantly generate a very large number of useful gene-specific primers from a small library of simple precursors. PMOS can be used to generate primers directly in the end-users laboratory within the context of any DNA polymerase chemistry such as in PCR or sequencing reactions

**Results:**

To demonstrate the utility of PMOS, a universal 768-member oligonucleotide library (UniSeq) was designed, fabricated and its performance optimized and evaluated in a range of PCR and DNA sequencing reactions. This methodology used to derive specific 11-mers, performed well in each of these activities and produced the desired amplification or sequencing analysis with results comparable to primers made by time consuming and expensive custom synthesis.

**Conclusion:**

On the basis of these experiments, we believe this novel system would be broadly applicable and could in many circumstances replace the need for conventional oligonucleotide synthesis.

## Background

In the last twenty years there has been an explosion in the demand for custom oligonucleotide synthesis to support a vast array of genomics applications. Despite improvements and automation in standard solid phase phosphoramidite chemistry, oligonucleotide synthesis remains a relatively inefficient, expensive and wasteful process, where the scale of synthesis may be many thousands of times the minute quantities required for a single PCR or sequencing reaction. Cost considerations have led to most oligonucleotides being synthesized at dedicated off-site facilities or large core facilities. The extra transportation time has hindered the development of applications requiring rapid turn around times.

These undesirable aspects of custom oligonucleotide synthesis, can be avoided by employing a pre-synthesized shortmer library in which each oligonucleotide is immediately available with little waste as only the primer needed is used[1,2]. Existing approaches, however, have proven of little utility as the primers from shortmer libraries are compromised by the necessity of constraining the library size to a manageable level. For example, while complete libraries of 5-mers and 6-mers (containing 1024 and 4096 oligonucleotides respectively), can be produced and used without difficulty, they lack the specificity and hybridization stability to work reliably as DNA polymerase primers[1]. Since each additional base increases the library size four fold, the shortest reliable primer length (10 or 11 bases) requires impossibly large libraries of 1 to 2 million primers.

In a compromise, partial libraries consisting of primers of 8 or 9 base length, have been designed for application in DNA sequencing [2-4]. These libraries have achieved little practical utility largely due to their poor priming efficiency on DNA templates containing significant secondary structure at the low hybridization temperatures required to anneal shortmers[1,5].

A number of refinements to this approach have attempted to overcome this deficiency by combining multiple primers to fabricate longer oligonucleotides with a defined sequence. In one method strings of two, three or more hexamers are assembled and ligated side by side on template DNA to form a primer[3,5-7]. More recently this has been accomplished with chemically ligated octamers [8]. Alternatively, unligated strings of 2-3 hexamers hybridize side by side to form a modular sequencing primer stabilized by base stacking interactions alone [9-13]. In another variation, the selected hexamers are ligated into defined strings by complimentary hexamers that overlap the junction in the opposite strand of a double helix [14]. While these developments of the shortmer strategy are appealing, their utility remains limited by poor hybridization and ligation efficiency, particularly on highly structured DNA templates that exist at the low temperatures required for hybridization and ligation of short modules [5].

In the methodology described here, we have developed an efficient composite primer strategy that uses DNA polymerase rather than DNA ligase to increase the hybridization affinity of library oligonucleotides. The priming specificity encoded by the unique pentamer sequence in each library member is more than doubled in a combinatorial process that uses one oligonucleotide as a template for polymerase extension. After careful consideration and optimization of this concept, a library consisting of 768 oligonucleotides was produced that can generate over 131,000 sequence optimized 11-mers. We have demonstrated that this DNA polymerase dependent assembly process can be seamlessly integrated into existing DNA sequencing reactions and provides all the advantages of using a preexisting primer library while maintain the hybridization specificity of chemical synthesis. While this approach was developed to facilitate the primer walking strategy for large scale DNA sequencing projects[15], this instant primer assembly system could have a plethora of applications in genomics and molecular biology.

## Results

### Polymerase mediated oligonucleotide synthesis (PMOS)

The PMOS system provides an efficient means to generate a large number of specific oligonucleotides from a small-prefabricated library of precursors. This is achieved by using one oligo from the library as a template for extension (in the presence of DNA polymerase) from the 3' end of another oligo in the library, such that the specificity encoded by each precursor is combined in the extended oligo.

**Figure 1:**
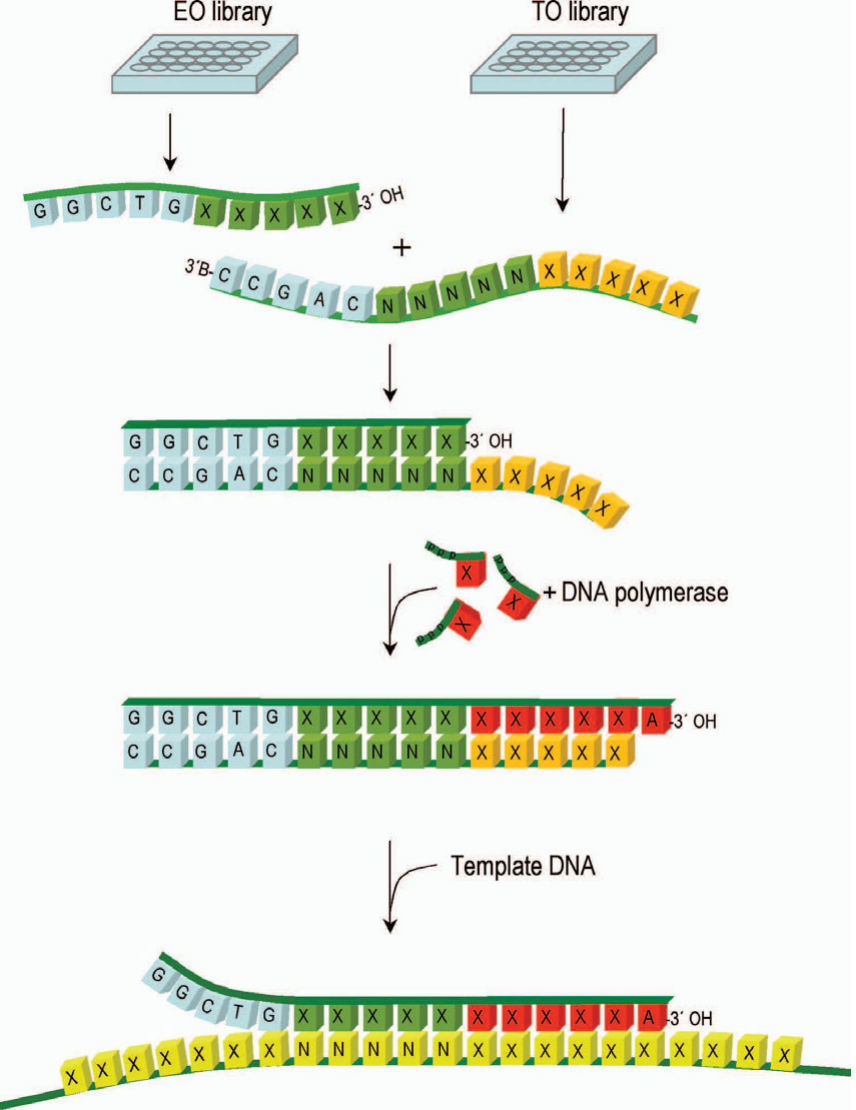
**PMOS primer assembly scheme**. The PMOS system consists of 256 extendable oligonucleotides (EO's) and 512 template oligonucleotides (TO's) distributed into two 384 well microtitre plates. In each case defined sequence positions for the individual library members are designated X; and in the TO degenerate positions are designated N. To generate each composite 11-mer, the required EO and TO is selected from the library and transferred to a reaction vessel. When combined the EO and TO associate via their complementary clamp (blue) and catch (green) regions. In the presence of DNA polymerase and nucleotides (orange), such as in sequencing chemistry, the EO is extended along the overlapping template (TO). Extension culminates with a single base extension (dA) beyond the limit of the template. In subsequent thermal cycles the sequence specific 11-mer derived from the extended EO can function as a template specific primer in sequencing and other applications. At the bottom of the diagram the composite 11-mer primer is shown annealed to template (yellow) as it finds deployment in the initiation of an extension reaction such as in DNA sequencing.

The precursor oligonucleotides that constitute the library are divided into template oligos (TO's) and extendable oligos (EO'S). The TO's contain a 3'-amine blocking group to prevent extension, whereas the extendable oligos (EO's) retain the capacity for extension by DNA polymerase and are in effect mini-primers (Fig. 1). Hybridization between the TO and EO occurs at a 10 bp overlap consisting of a 5 bp region of generic complementarity known as the "clamp" region and a 5 bp section termed the "catch" region. While the sequence in the catch region of each EO is unique, the corresponding region in the TO is degenerate to enable each one to hybridise with any EO (Fig. 1). This capacity for universal association between EO's and TO's enables the oligonucleotide library to mix in different combinations and produce a total number of different primers equal to the product of EO's and TO's (ΣS = ΣEO × ΣTO). The strength and alignment of the EO/TO interaction is supported by a defined GC rich clamp sequence (GGCTG with respect to the EO). This sequence is retained in the 5' end of each primer generated but does not affect their function in DNA sequencing and amplification.

### PCR using PMOS

**Figure 2:**
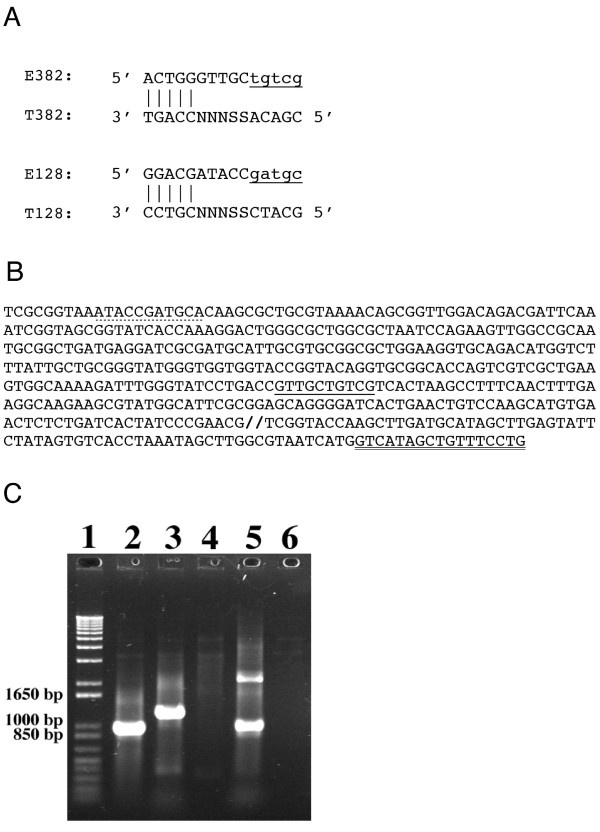
**DNA amplification using PMOS**. Panel A contains the design of EO's and TO's that combine to form the forward primers for amplification of the ftsZ gene. Vertical bars indicate clamp regions of hybridisation between the EO and TO. Capital letters show actual sequence of the oligonucleotides and small, underlined letters indicate the extended region of the EO. N represents a position with either A, T, G or C. S represents a position with either G or C nucleotide. Panel B contains the DNA template sequence of *E. coli *ftsZ and includes the recognition sequence for the M13 reverse primer (double underlined) within the plasmid pFC1. The position of the forward primers (formed by extended EO's) is indicated by the dotted and solid underlining. Panel C contains a photograph of a 1% agarose gel produced after amplification of the ftsZ sequence. Lane 1 contained a DNA molecular weight marker with corresponding sizes shown on the left. Lane 2 contains an amplification reaction with oligonucleotides EC10 and M13 reverse (positive control). Lane 3 contains an amplification reaction with oligonucleotides E128, T128 and M13 reverse. Lane 4 contains an amplification reaction with oligonucleotides E128 and M13 reverse (negative control). Lane 5 contains an amplification reaction with oligonucleotides E382, T382 and M13 reverse. Lane 6 contains an amplification reaction with oligonucleotides E382 and M13 reverse (negative control).

The application of PMOS in PCR was shown in two separate reactions carried out using the forward primer pairs E128/T128 and E382/T382 (Fig. 2A). In each reaction the conventional M13 reverse primer was used on a 4.6 kilobase plasmid (pFC1) containing the *ftsZ *gene insert from *E. coli *template DNA (Fig. 2B). Amplification was carried out over 32 cycles and the products resolved on a 1% agarose gel. PCRs containing EO's 128 or 382 and M13 reverse alone were incapable of generating PCR products (Fig. 2C, lanes 4 and 6). However, when E128, T128, and E382, T382 were all present, the expected PCR amplicons of length 1165 and 911 respectively were obtained (Fig. 2C, lanes 3 and 5). A positive control reaction was performed using EC10, a full-length synthetic primer equivalent to the extended E382 (Fig. 2C, lanes 2).

While both composite forward primers generated their respective amplicons with M13 reverse, we found that the E128/T128 pair displayed greater specificity and efficiency than that provided by E382/T382. We hypothesised that this was due to the non-template dependent addition of a single adenosine to the 3' end of the EO beyond the extremity of its TO. In the case of E128/T128 pair, this A addition provides an extra nucleotide that assists in hybridization with a corresponding T in the target sequence, thus bringing its annealing length to 11 bases. This would enhance both the affinity and specificity of the extended primer. By contrast, the addition of an A at the end of the extended E382/T382 pair produces an 11-mer that is terminally mismatched with respect to the target template sequence.

### DNA cycle sequencing using PMOS

**Figure 3:**
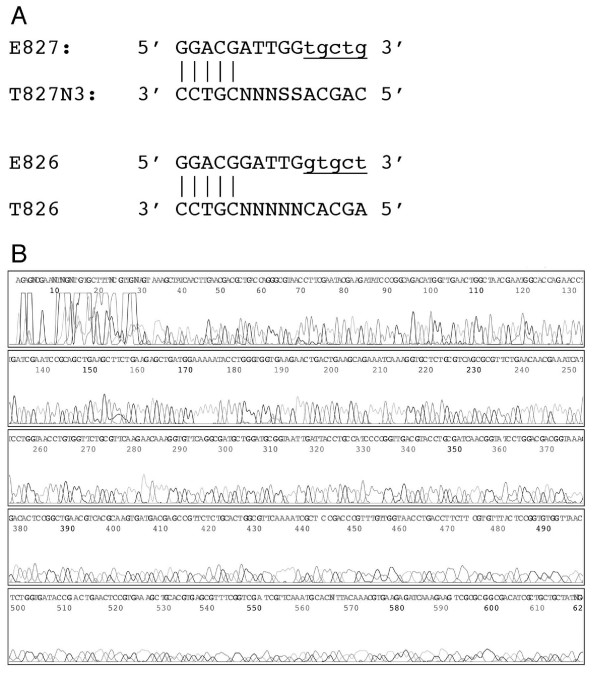
**DNA cycle sequencing using an extendable primer**. Panel A contains the design of an EOs and TOs for the sequencing of a region of the *Escherichia coli *streptomycin operon. Vertical bars indicate regions of clamp region of hybridisation between the EO and TO. Capital letters show actual sequence of the oligonucleotides and small, underlined letters indicate the extended region of the EO. N indicates a position with either A, T, G or C and S indicates a position with either G or C nucleotide. Panel B shows an electropherogram of a DNA sequencing reaction using E827 and T827N3. The sequencing reaction was separated and analyzed on an ABI PRISM 377 DNA sequencer and ABI PRISM sequence analysis software.

To examine the potential of PMOS for DNA sequencing, oligonucleotide E827 and template oligonucleotide T827N3 (Fig. 3A) were mixed with a linear DNA fragment from the streptomycin operon in *E. coli*, 4 μl of BigDye sequencing chemistry, 1 μl of 17.5 mM MgCl_2 _and 1 μl of 300 μM dGTP. After 40 thermocycles, the sequencing fragments were resolved on an ABI 377 DNA sequencer and analysed by ABI PRISM™ sequence analysis software. The electropherogram from this reaction displayed strong signal strength and the expected sequence (Fig. 3B). A negative control reaction primed by the non-extended E827 primer was performed but no sequence signal was generated.

### Optimisation of sequencing chemistry for PMOS

The previous experiment demonstrated that a combination of extendable oligonucleotides and template oligonucleotides with a degenerate catch region could be used directly to prime DNA sequencing reactions. During this investigation we found that reactions in commercial sequencing chemistries were enhanced by supplementing with MgCl_2 _and dGTP. The optimal magnesium chloride concentration was determined through a series of sequencing reactions performed with the addition of 1 μl of 0, 7.5, 12.5, 17.5, 22.5, 25, 30, 40 and 50 mM MgCl_2 _solution. The best result was achieved after adding the 17.5 mM solution. At lower concentrations there was a reduction of sequence signal, while at higher concentrations there was no further improvement (data not shown).

We also found that additional dGTP improved sequencing signal strength using the PMOS primers in ABI BigDye sequencing chemistry version 2. After testing a range of dGTP concentrations between 0 and 50 μM, 30 μM supplement was found to be optimal. At higher concentrations there was an increase in sequencing errors, while lower concentrations reduced the sequencing signal. We also looked at other commercial sequencing chemistries such as ABI BigDye sequencing chemistry version 3 and DYEnamic ET Terminator (GE Bioscience). Both of these chemistries were compatible with direct PMOS primer assembly and yielded good sequencing results without any further modification of the PMOS method (data not shown).

The optimal EO:TO molar ratio was determined by varying the concentration of T827N3 from 0.25 to 8 μM, while keeping the E827 concentration constant at 1 μM. An EO to TO molar ratio of between 1:1 and 2:1 was found to give the highest quality sequencing results. Higher EO:TO ratios were found to result in less signal intensity, presumably due to inefficient extension of the EO primer in the presence of limiting amounts of the TO primer. Lower ratios (i.e. excess TO) gave mixed sequence signal.

The optimal concentrations of EO and TO primers in the sequencing reaction were also determined. The E827 and T827N3 concentration (at a 1:1 ratio) were varied between 0.25 and 8 μM. The optimum concentration was found to be 1 μM. Lower concentrations produced high quality sequence at the expense of reduced signal intensity. Higher primer concentrations produced more sequencing signal, but at the expense of an increased error rate (data not shown).

### Optimisation of PMOS oligonucleotides

**Figure 4:**
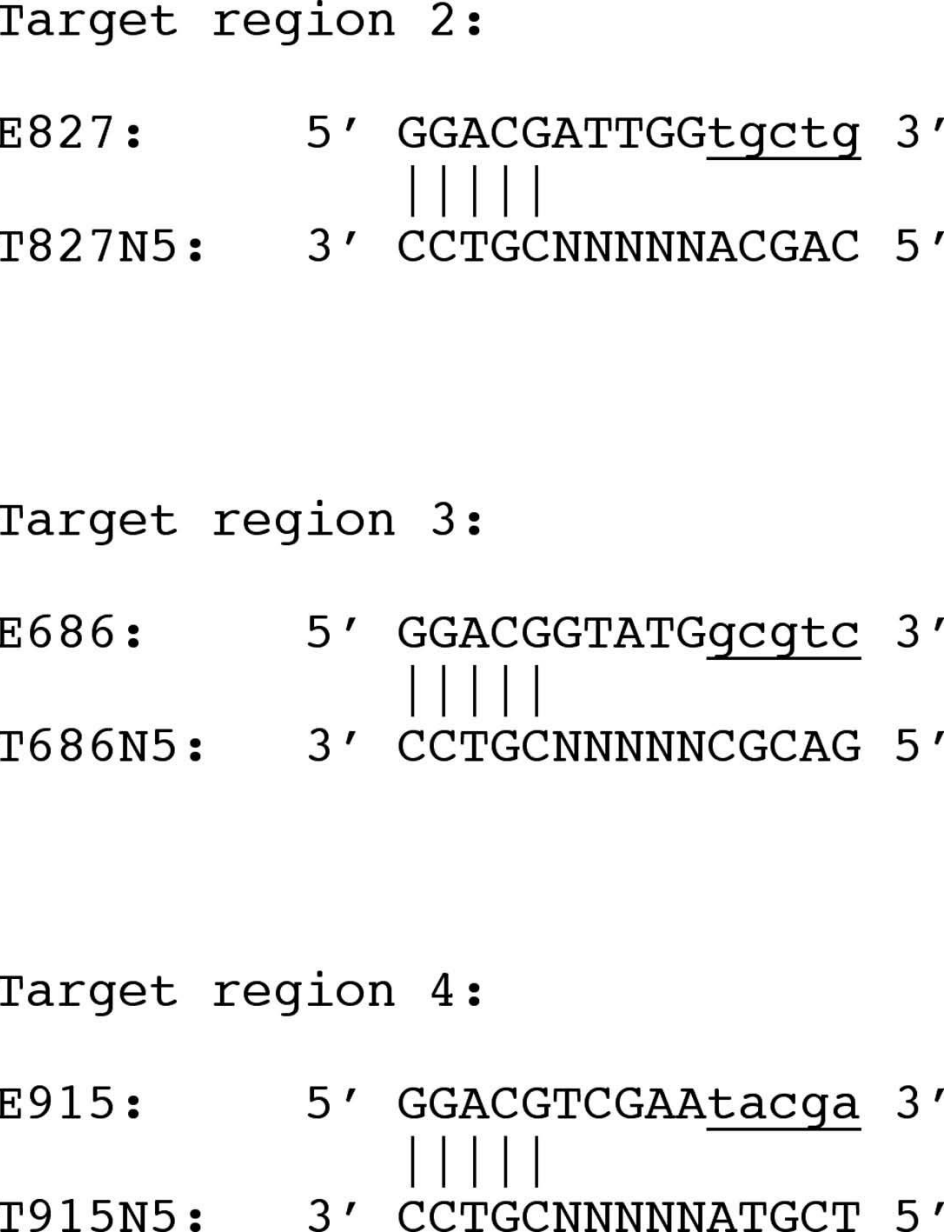
**Design of EOs and TOs for sequencing a region of the E. coli streptomycin operon**. Region 2, 3 and 4 primers have decreasing levels GC content in the 3' terminal of the EO catch.

A key feature of the PMOS library system (UniSeq) is the ability of the unique 5 base sequences in the catch region of each EO primer to hybridise with the corresponding generic region in every TO primer (Figure 1). This is accomplished with a degenerate or mixed base composition in the catch region of the TO. To determine the influence of catch region hybridisation strength on sequencing quality, three sequencing reactions were performed using three different EO/TO pairs with ascending levels of G+C content (Fig. 4). As predicted the EO primer with the highest 5'-G+C content (E827) produced the most sequencing signal, followed by intermediate (E686), and no G+C content (E915) respectively (data not shown).

**Figure 5:**
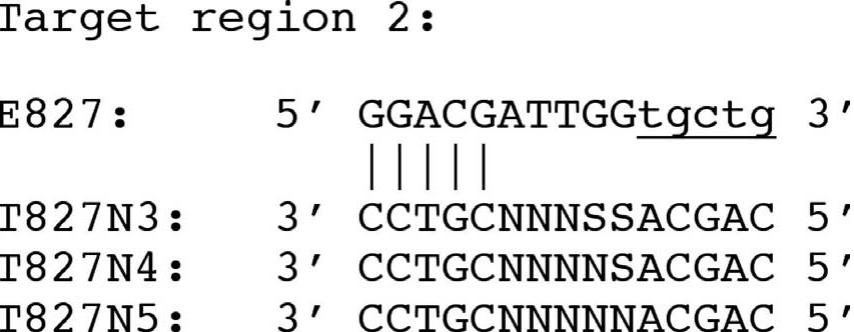
**Design of E827 and cognate TOs with escalating levels of catch region degeneracy**. These primers were used to sequence a region of the *E. coli *streptomycin operon.

In order to accommodate the observed preference for high G+C content in this part of the catch region we restricted the EO library to oligonucleotides that contained G or C bases at positions 9 & 10. This also allowed for the reduction in degeneracy at the corresponding position in TOs. An experiment was performed to investigate the impact of this change by comparing sequencing efficiency of complete TO catch degeneracy (NNNNN) with TOs having reduced degeneracy at one position (SNNNN) and two positions (SSNNN) where S is an equal mixture of G and C (Fig. 5). As expected, T827N3 and T827N4 resulted in greater DNA sequencing signals than the completely degenerate version T827N5 (data not shown).

To further maximise catch region hybridisation efficiency, the adenosine bases (when they appear in the first 3 positions) of the EO were substituted for the high affinity analogue 2,6-diaminopurine (D) [16]. This substitution was found to further improve DNA sequencing signal and efficiency.

### Effect of non-template addition of adenosine in PMOS

DNA sequencing protocols typically employ DNA polymerases lacking 3'-5' exonuclease and have a tendency to add non-template directed adenine residue at the 3' end of extension product[17]. As a consequence an EO primer extended with a DNA sequencing polymerase will usually have an additional 3' adenine. Primers with this additional 3' adenine are not expected to function effectively in sequencing reaction unless there is a corresponding thymine on the template sequence. To test this hypothesis, EO and TO primers were designed for a target site that did not contain a complementary thymine downstream of the target site (one base upstream of E827). A cycle sequencing reaction was performed as described previously with 10 pmol of E826 and 10 pmol of T626 (Fig 3A). Only very poor sequencing data was obtained, which indicates that an additional 3'A on an extended EO without a complementary position in the sequencing template prevents efficient extension during the sequencing reaction.

### DNA cycle sequencing using PMOS library primers

**Figure 6:**
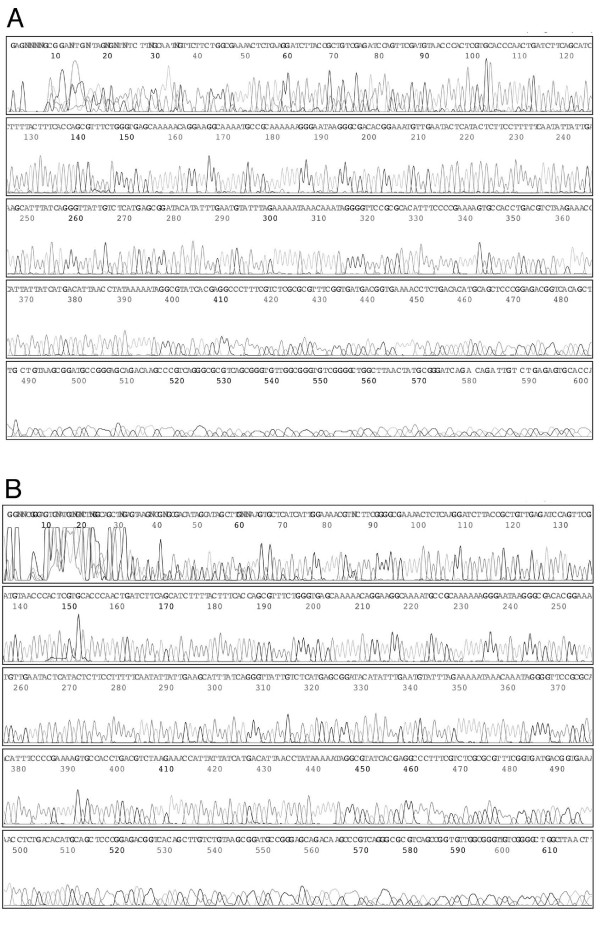
**DNA sequencing reactions using PMOS primers**. Panels A and B contain an electropherograms of a DNA sequencing reactions using library derived E154/T422 and E167/T14 respectively. The sequencing reaction was separated and analyzed on an ABI PRISM 377 DNA sequencer and ABI PRISM sequence analysis software.

A biologically optimised library consisting of 256 extendable oligonucleotides and 512 compatible template oligonucleotides was synthesised for the purpose of testing PMOS in sequencing projects (Additional file 1 and 2). The PMOS library, distributed across two 384 well plates, has been used successfully in our laboratory in thousands of DNA sequencing reactions. This is exemplified here in two reactions on pUC19 template DNA carried out using EO/TO pairs E154/T422 and E167/T14, respectively, according to conditions described earlier. The electropherograms from both reactions were strong and gave an unambiguous signal corresponding to the expected sequence in each case (Fig. 6). To validate the utility for PMOS in cycle sequencing, we carried out 1344 sequencing reaction of a BAC library without any specific optimisation and achieved a success rate of 65% (Q20>100 bp). While this success rate was lower than that produced for an equivalent shotgun project, possibly due in part to the failure of some PMOS primers, the overall coverage achieved by this approach was superior and required substantially fewer sequencing reactions. Gaps produced by as a consequence of failed reaction were closed using adjacent PMOS primers targeting upstream and downstream segments of the template DNA.

## Discussion

The universal primer fabrication system (PMOS) described here provides a simple and efficient means to generate specific 11-mer from a 768-member library of pre-synthesised oligonucleotides. In the PMOS approach, the oligonucleotide of interest is generated by hybridising two precursors, such that in the presence of a DNA polymerase (such as in DNA sequencing and amplification reactions), the desired primer is generated by an extension reaction (Fig. 1). To maximize the utility of PMOS, we examined the influence of a number of parameters in order to generate the optimal library of EO and TO precursors molecules.

### Optimisation of PMOS oligonucleotide design

The versatility of PMOS is derived from its ability to achieve both high specificity and high coverage in a very small library. To find the ideal balance between the conflicting demands of specificity and simplicity, the hybridisation kinetics of the EO/TO pair and S-primer/target pair was finely tuned while managing the library complexity. The key strategy employed to reduce the library size was to introduce degeneracy into the catch region of the TO such that it will have complementarity and hybridise with each and every EO. This meant that with only 256 EOs and 512 TOs the library could generate 131,072 (256 × 512) different extended EOs. Rather than introduce complete sequence redundancy at all five catch positions, we restricted the 5' dinucleotide of the catch region TOs to an equal mixture of guanine and cytosine (Fig. 3A). The corresponding variable region at the 3' terminus of the EOs was also restricted to the four different combinations of guanine and cytosine to enforce high affinity interaction with each TO at the point of extension.

The use of the generic bases 5-nitroindole and 3-nitropyrole was investigated as an alternative to degenerate positions in the TO catch region. While these universal nucleotide analogues are reported to be capable of binding with all four conventional bases, we found their performance inferior to degeneracy. A modification of the catch region that we did find effective, was the replacement of adenosine with its high affinity analogue 2,6-diaminopurine [16,18]. This base analogue has the ability to form tridentate hydrogen bonds with thymine, increasing both the hybridisation strength between EO/TO and the S-primer to its target sequence (data not shown).

A number of different designs for the clamp region were also considered and tested to maximise the hybridisation strength while minimising interaction with motifs commonly found in cloning vectors and genomes. The clamp region sequence that was used (GGACG) fulfilling these requirements with a low free energy of hybridisation and no sequence similarity to plasmid backbones of the common pUC plasmid family.

### Library rationalisation

To reduce the size of the library, sequence motifs that are not found, or that are very rare in biological systems, were omitted. The library was also screened for sequences with difficult motifs characterised by extreme GC content; monotony such as polynucleotide runs; form primer dimers or hairpin formation. Removal of these EOs and TOs resulted in a substantial reduction in library complexity without significantly lowering its utility for hybridizing to biologically derived nucleic acid templates.

To further enhance the utility of the library we developed companion software (UniSelect) to facilitate the primer selection process. This software scans within the target range of input sequences and identifies the best TO and EO pair(s) from the library for the purpose of amplifying the target or sequencing the upstream or downstream template. The output of UniSelect can be used directly as an input for liquid handling robots to complete the cycle from sequence data collection back to reaction setup, without the need for human intervention.

### Non-template directed extension

The DNA polymerases commonly used for PCR and sequencing such as *Taq *DNA polymerase and Thermosequenase catalyse non-template directed addition of a single nucleotide. While the extent of this activity is influenced to some degree by the terminal sequence [19,20], the extended nucleotide is almost always adenosine because of its higher affinity for the active site in the absence of template [17,21]. In our experience extendable primers designed without regard to this affect were severely impaired in their ability to prime sequencing and amplification reactions. This was likely due to the terminal mismatch between these primers and their template sequence. By contrast extendable primers selected to take advantage of this effect by pairing this extra base with a corresponding thymine in the template were far superior and behaved as 11-mers. However, in circumstances where terminal addition of adenosine is unwanted, a proofreading polymerase can be used to ensure strict template directed extension [17]. We achieve this routinely by performing PMOS in the presence of Klenow fragment.

### Applications

While solid phase chemical synthesis methods can provide oligonucleotides for a myriad of biomolecular and nanotechnology applications, the process is relatively slow and wasteful where only small quantities are required. PMOS is an efficient primer construction system that is capable of generating a large number of high affinity oligonucleotides from a small library of prefabricated precursors. The system features a library consisting of 768 oligonucleotides that generates more than 131,000 different biologically optimized 11-mers via an efficient primer extension reaction. This contrasts with the hexamer and octamer libraries, which are both complex (4096 and 65,536 primers respectively) and inefficient.

While we have focussed primarily cycle sequencing, PMOS can potentially be used in any application requiring oligonucleotides including, ligation chain reaction (LCR) [22], reverse-transcriptase PCR (RT-PCR) [23], primer extension reaction for mRNA-transcript analysis[24], self-sustaining sequence replication [25], rolling circle amplification[26], strand displacement amplification [27], isothermal DNA amplification[28] and DNA-sequencing by the original Sanger method [29]. For applications that do not require DNA polymerase activity, extension of EOs can be performed prior to use in a preparative reaction.

## Conclusion

The PMOS library system coupled with extension mediated primer synthesis provides the ability to efficiently obtain primers in minutes without waste and at a fraction of the cost of custom solid phase synthesis. This has many applications including the potential to replace shotgun DNA sequencing with a more rational primer walking strategy, with up to 80% savings in resources, time and cost.

## Methods

The PMOS oligonucleotide library was organized into two 384 well microtitre plates consisting of 256 extendible oligonucleotides and 512 template oligonucleotides. These oligonucleotides were manufactured according to the sequences list in Additional files 1 and 2 by Sigma Genosys (The Woodlands, TX).

### Primer extension and target amplification

S-primer synthesis was carried out directly within the PCR as follows. Plasmid template pFC1 (1 ng) was combined with 1 μl of the E128 or 382 (10 pmol/μl), one μl of the T128 or 382 (20 pmol/μl), 1 μl of M13 reverse primer (5'-CAGGAAACAGCTATGAC-3'; 5 pmol/μl), 2 μl of 25 mM MgCl_2_, 4 μl of 1 mM dNTPs (MBI Fermentas, Vilnius, Lithuania), 2 μl of 10 × buffer [100 mM Tris-HCl (pH 9 at 25°C), 500 mM potassium chloride (KCl), 1% (v/v) Triton X-100 (Promega)], and water to final volume of 16 μl. For the negative control reaction the TO primer was omitted. For the positive control the EO and TO were replaced by the control primer EC10 (5' GTTGCTGTCG 3') targeting the same region (10 pmol). The mixture was heated for one min at 95°C and then cooled to 80°C before addition of 4 μl of *Taq *DNA polymerase (0.25 units/μl; Promega). The reactions were then cycled 32 times at 95°C for 10 sec, at 51°C for 20 sec and at 72°C for 1.5 min. After a final incubation at 72°C for 5 min the reactions were stored at 4°C before electrophoresis on a 1% (w/v) agarose gel. Amplicons were visualised by staining in ethidium bromide.

### DNA cycle sequencing with PMOS primers

Sequencing reactions were performed using BigDye sequencing system version 2 (Applera Corporation, Norwalk, CT USA) supplemented with additional magnesium chloride and dGTP. The optimal conditions contained 10 pmol of EO, 10 pmol of TO, 100-300 ng of template DNA, 1 μl of 17.5 mM MgCl_2_, 1 μl of 300 μM dGTP, 4 μl of BigDye sequencing reagent, and water to a final volume of 10 μl. The reaction was then cycled 40 times at 96°C for 10 sec, at 45°C for 30 sec and at 60°C for 4 min before purification by butanol extraction [30]. The cleaned sequencing reaction were analyzed on an ABI PRISM 377 DNA sequencer using the ABI PRISM sequence analysis software (Applera Corp., Norwalk, CT, USA) according to the manufacturer's instructions.

## Abbreviations

PMOS: polymerase mediated oligonucleotide synthesis; TO: template oligonucleotide; EO: extendable oligonucleotide; PCR: polymerase chain reaction.

## Authors' contributions

DT conceived the PMOS methodology and designed the system. TT and CB performed the experiments. MC prepared the manuscript.

## Supplementary Material

Additional file 1 **A library of extendable oligonucleotides (EOs)**. Table containing extendable oligonucleotide sequences. Click here for file

Additional file 2 **A library of template oligonucleotides (TOs)**. Table containing template oligonucleotide sequences. Click here for file
