# Supplementary material for: Primer fabrication using polymerase mediated oligonucleotide synthesis
Source: BMC Genomics. 2009 Jul 31;10:344. doi: 10.1186/1471-2164-10-344 (PMC2733156; doi:10.1186/1471-2164-10-344)
Supplement: Additional file 1 — A library of extendable oligonucleotides (EOs). Table containing extendable oligonucleotide sequences. [file 1471-2164-10-344-S1.doc]

**Table 1: A library of extendable oligonucleotides (EOs).** The left column shows a oligonucleotide identification code and the right column shows the sequence 5’ to 3’ from left to right. “D” stands for 2,4 diamino purine.

| EO-ID | Sequence (5’ to 3’) |
| --- | --- |
| E001-DDDCC | CGTCCDDDCC |
| E002-DDCCC | CGTCCDDCCC |
| E003-DDGCC | CGTCCDDGCC |
| E004-DDTCC | CGTCCDDTCC |
| E005-DCDCC | CGTCCDCDCC |
| E006-DCCCC | CGTCCDCCCC |
| E007-ACGCC | CGTCCACGCC |
| E008-DCTCC | CGTCCDCTCC |
| E009-DGDCC | CGTCCDGDCC |
| E010-AGCCC | CGTCCAGCCC |
| E011-AGGCC | CGTCCAGGCC |
| E012-DGTCC | CGTCCDGTCC |
| E013-DTDCC | CGTCCDTDCC |
| E014-DTCCC | CGTCCDTCCC |
| E015-DTGCC | CGTCCDTGCC |
| E016-DTTCC | CGTCCDTTCC |
| E017-CDDCC | CGTCCCDDCC |
| E018-CDCCC | CGTCCCDCCC |
| E019-CAGCC | CGTCCCAGCC |
| E020-CDTCC | CGTCCCDTCC |
| E021-CCACC | CGTCCCCACC |
| E022-CCCCC | CGTCCCCCCC |
| E023-CCGCC | CGTCCCCGCC |
| E024-CCTCC | CGTCCCCTCC |
| E025-CGDCC | CGTCCCGDCC |
| E026-CGCCC | CGTCCCGCCC |
| E027-CGGCC | CGTCCCGGCC |
| E028-CGTCC | CGTCCCGTCC |
| E029-CDTCC | CGTCCCDTCC |
| E030-CTCCC | CGTCCCTCCC |
| E031-CTGCC | CGTCCCTGCC |
| E032-CTTCC | CGTCCCTTCC |
| E033-GDDCC | CGTCCGDDCC |
| E034-GDCCC | CGTCCGDCCC |
| E035-GDGCC | CGTCCGDGCC |
| E036-GDTCC | CGTCCGDTCC |
| E037-GCDCC | CGTCCGCDCC |
| E038-GCCCC | CGTCCGCCCC |
| E039-GCGCC | CGTCCGCGCC |
| E040-GCTCC | CGTCCGCTCC |
| E040-GCTCC | CGTCCGCTCC |
| E041-GGDCC | CGTCCGGDCC |
| E042-GGCCC | CGTCCGGCCC |
| E043-GGGCC | CGTCCGGGCC |
| E044-GGTCC | CGTCCGGTCC |
| E045-GTDCC | CGTCCGTDCC |
| E046-GTCCC | CGTCCGTCCC |
| E047-GTGCC | CGTCCGTGCC |
| E048-GTTCC | CGTCCGTTCC |
| E049-TDDCC | CGTCCTDDCC |
| E050-TDCCC | CGTCCTDCCC |
| E051-TDGCC | CGTCCTDGCC |
| E052-TDTCC | CGTCCTDTCC |
| E053-TCDCC | CGTCCTCDCC |
| E054-TCCCC | CGTCCTCCCC |
| E055-TCGCC | CGTCCTCGCC |
| E056-TCTCC | CGTCCTCTCC |
| E057-TGDCC | CGTCCTGDCC |
| E058-TGCCC | CGTCCTGCCC |
| E059-TGGCC | CGTCCTGGCC |
| E060-TGTCC | CGTCCTGTCC |
| E061-TTDCC | CGTCCTTDCC |
| E062-TTCCC | CGTCCTTCCC |
| E063-TTGCC | CGTCCTTGCC |
| E064-TTTCC | CGTCCTTTCC |
| E065-DDDCG | CGTCCDDDCG |
| E066-DDCCG | CGTCCDDCCG |
| E067-DDGCG | CGTCCDDGCG |
| E068-DDTCG | CGTCCDDTCG |
| E069-DCDCG | CGTCCDCDCG |
| E070-DCCCG | CGTCCDCCCG |
| E071-DCGCG | CGTCCDCGCG |
| E072-DCTCG | CGTCCDCTCG |
| E073-DGDCG | CGTCCDGDCG |
| E074-DGCCG | CGTCCDGCCG |
| E075-DGGCG | CGTCCDGGCG |
| E076-DGTCG | CGTCCDGTCG |
| E077-DTDCG | CGTCCDTDCG |
| E078-DTCCG | CGTCCDTCCG |
| E079-DTGCG | CGTCCDTGCG |
| E080-DTTCG | CGTCCDTTCG |
| E081-CDDCG | CGTCCCDDCG |
| E082-CDCCG | CGTCCCDCCG |
| E083-CDGCG | CGTCCCDGCG |
| E084-CDTCG | CGTCCCDTCG |
| E085-CCDCG | CGTCCCCDCG |
| E086-CCCCG | CGTCCCCCCG |
| E087-CCGCG | CGTCCCCGCG |
| E088-CCTCG | CGTCCCCTCG |
| E089-CGDCG | CGTCCCGDCG |
| E090-CGCCG | CGTCCCGCCG |
| E091-CGGCG | CGTCCCGGCG |
| E092-CGTCG | CGTCCCGTCG |
| E093-CTDCG | CGTCCCTDCG |
| E094-CTCCG | CGTCCCTCCG |
| E095-CTGCG | CGTCCCTGCG |
| E096-CTTCG | CGTCCCTTCG |
| E097-GDDCG | CGTCCGDDCG |
| E098-GDCCG | CGTCCGDCCG |
| E099-GAGCG | CGTCCGAGCG |
| E100-GDTCG | CGTCCGDTCG |
| E101-GCDCG | CGTCCGCDCG |
| E102-GCCCG | CGTCCGCCCG |
| E103-GCGCG | CGTCCGCGCG |
| E104-GCTCG | CGTCCGCTCG |
| E105-GGDCG | CGTCCGGDCG |
| E106-GGCCG | CGTCCGGCCG |
| E107-GGGCG | CGTCCGGGCG |
| E108-GGTCG | CGTCCGGTCG |
| E109-GTDCG | CGTCCGTDCG |
| E110-GTCCG | CGTCCGTCCG |
| E111-GTGCG | CGTCCGTGCG |
| E112-GTTCG | CGTCCGTTCG |
| E113-TDDCG | CGTCCTDDCG |
| E114-TDCCG | CGTCCTDCCG |
| E115-TDGCG | CGTCCTDGCG |
| E116-TDTCG | CGTCCTDTCG |
| E117-TCDCG | CGTCCTCDCG |
| E118-TCCCG | CGTCCTCCCG |
| E119-TCGCG | CGTCCTCGCG |
| E120-TCTCG | CGTCCTCTCG |
| E121-TGDCG | CGTCCTGDCG |
| E122-TGCCG | CGTCCTGCCG |
| E123-TGGCG | CGTCCTGGCG |
| E124-TGTCG | CGTCCTGTCG |
| E125-TTDCG | CGTCCTTDCG |
| E126-TTCCG | CGTCCTTCCG |
| E127-TTGCG | CGTCCTTGCG |
| E128-TTTCG | CGTCCTTTCG |
| E129-DDDGC | CGTCCDDDGC |
| E130-DDCGC | CGTCCDDCGC |
| E131-DDGGC | CGTCCDDGGC |
| E132-DDTGC | CGTCCDDTGC |
| E133-DCDGC | CGTCCDCDGC |
| E134-DCCGC | CGTCCDCCGC |
| E135-DCGGC | CGTCCDCGGC |
| E136-DCTGC | CGTCCDCTGC |
| E137-DGDGC | CGTCCDGDGC |
| E138-DGCGC | CGTCCDGCGC |
| E139-DGGGC | CGTCCDGGGC |
| E140-DGTGC | CGTCCDGTGC |
| E141-DTDGC | CGTCCDTDGC |
| E142-DTCGC | CGTCCDTCGC |
| E143-DTGGC | CGTCCDTGGC |
| E144-DTTGC | CGTCCDTTGC |
| E145-CDDGC | CGTCCCDDGC |
| E146-CDCGC | CGTCCCDCGC |
| E147-CDGGC | CGTCCCDGGC |
| E148-CDTGC | CGTCCCDTGC |
| E149-CCDGC | CGTCCCCDGC |
| E150-CCCGC | CGTCCCCCGC |
| E151-CCGGC | CGTCCCCGGC |
| E152-CCTGC | CGTCCCCTGC |
| E153-CGDGC | CGTCCCGDGC |
| E154-CGCGC | CGTCCCGCGC |
| E155-CGGGC | CGTCCCGGGC |
| E156-CGTGC | CGTCCCGTGC |
| E157-CTDGC | CGTCCCTDGC |
| E158-CTCGC | CGTCCCTCGC |
| E159-CTGGC | CGTCCCTGGC |
| E160-CTTGC | CGTCCCTTGC |
| E161-GDDGC | CGTCCGDDGC |
| E162-GDCGC | CGTCCGDCGC |
| E163-GDGGC | CGTCCGDGGC |
| E164-GDTGC | CGTCCGDTGC |
| E165-GCDGC | CGTCCGCDGC |
| E166-GCCGC | CGTCCGCCGC |
| E167-GCGGC | CGTCCGCGGC |
| E168-GCTGC | CGTCCGCTGC |
| E169-GGDGC | CGTCCGGDGC |
| E170-GGCGC | CGTCCGGCGC |
| E171-GGGGC | CGTCCGGGGC |
| E172-GGTGC | CGTCCGGTGC |
| E173-GTDGC | CGTCCGTDGC |
| E174-GTCGC | CGTCCGTCGC |
| E175-GTGGC | CGTCCGTGGC |
| E176-GTTGC | CGTCCGTTGC |
| E177-TDDGC | CGTCCTDDGC |
| E178-TDCGC | CGTCCTDCGC |
| E179-TDGGC | CGTCCTDGGC |
| E180-TDTGC | CGTCCTDTGC |
| E181-TCDGC | CGTCCTCDGC |
| E182-TCCGC | CGTCCTCCGC |
| E183-TCGGC | CGTCCTCGGC |
| E184-TCTGC | CGTCCTCTGC |
| E185-TGDGC | CGTCCTGDGC |
| E186-TGCGC | CGTCCTGCGC |
| E187-TGGGC | CGTCCTGGGC |
| E188-TGTGC | CGTCCTGTGC |
| E189-TTDGC | CGTCCTTDGC |
| E190-TTCGC | CGTCCTTCGC |
| E191-TTGGC | CGTCCTTGGC |
| E192-TTTGC | CGTCCTTTGC |
| E193-DDDGG | CGTCCDDDGG |
| E194-DDCGG | CGTCCDDCGG |
| E195-DDGGG | CGTCCDDGGG |
| E196-DDTGG | CGTCCDDTGG |
| E197-DCDGG | CGTCCDCDGG |
| E198-ACCGG | CGTCCACCGG |
| E199-ACGGG | CGTCCACGGG |
| E200-DCTGG | CGTCCDCTGG |
| E201-DGDGG | CGTCCDGDGG |
| E202-AGCGG | CGTCCAGCGG |
| E203-AGGGG | CGTCCAGGGG |
| E204-DGTGG | CGTCCDGTGG |
| E205-DTDGG | CGTCCDTDGG |
| E206-DTCGG | CGTCCDTCGG |
| E207-DTGGG | CGTCCDTGGG |
| E208-DTTGG | CGTCCDTTGG |
| E209-CDDGG | CGTCCCDDGG |
| E210-CDCGG | CGTCCCDCGG |
| E211-CAGGG | CGTCCCAGGG |
| E212-CDTGG | CGTCCCDTGG |
| E213-CCDGG | CGTCCCCDGG |
| E214-CCCGG | CGTCCCCCGG |
| E215-CCGGG | CGTCCCCGGG |
| E216-CCTGG | CGTCCCCTGG |
| E217-CGAGG | CGTCCCGAGG |
| E218-CGCGG | CGTCCCGCGG |
| E219-CGGGG | CGTCCCGGGG |
| E220-CGTGG | CGTCCCGTGG |
| E221-CTDGG | CGTCCCTDGG |
| E222-CTCGG | CGTCCCTCGG |
| E223-CTGGG | CGTCCCTGGG |
| E224-CTTGG | CGTCCCTTGG |
| E225-GDDGG | CGTCCGDDGG |
| E226-GDCGG | CGTCCGDCGG |
| E227-GDGGG | CGTCCGDGGG |
| E228-GDTGG | CGTCCGDTGG |
| E229-GCDGG | CGTCCGCDGG |
| E230-GCCGG | CGTCCGCCGG |
| E231-GCGGG | CGTCCGCGGG |
| E232-GCTGG | CGTCCGCTGG |
| E233-GGDGG | CGTCCGGDGG |
| E234-GGCGG | CGTCCGGCGG |
| E235-GGGGG | CGTCCGGGGG |
| E236-GGTGG | CGTCCGGTGG |
| E237-GTDGG | CGTCCGTDGG |
| E238-GTCGG | CGTCCGTCGG |
| E239-GTGGG | CGTCCGTGGG |
| E240-GTTGG | CGTCCGTTGG |
| E241-TDDGG | CGTCCTDDGG |
| E242-TDCGG | CGTCCTDCGG |
| E243-TDGGG | CGTCCTDGGG |
| E244-TDTGG | CGTCCTDTGG |
| E245-TCDGG | CGTCCTCDGG |
| E246-TCCGG | CGTCCTCCGG |
| E247-TCGGG | CGTCCTCGGG |
| E248-TCTGG | CGTCCTCTGG |
| E249-TGDGG | CGTCCTGDGG |
| E250-TGCGG | CGTCCTGCGG |
| E251-TGGGG | CGTCCTGGGG |
| E252-TGTGG | CGTCCTGTGG |
| E253-TTDGG | CGTCCTTDGG |
| E254-TTCGG | CGTCCTTCGG |
| E255-TTGGG | CGTCCTTGGG |
| E256-TTTGG | CGTCCTTTGG |
