# Supplementary material for: Primer fabrication using polymerase mediated oligonucleotide synthesis
Source: BMC Genomics. 2009 Jul 31;10:344. doi: 10.1186/1471-2164-10-344 (PMC2733156; doi:10.1186/1471-2164-10-344)
Supplement: Additional file 2 — A library of template oligonucleotides (TOs). Table containing template oligonucleotide sequences. [file 1471-2164-10-344-S2.doc]

**Table 2: A library of template oligonucleotides (TOs)**. The left column shows a oligonucleotide identification code and the right column shows the sequence (5’ to 3’).

| TO-ID | Sequence (5’ to 3’) |
| --- | --- |
| T001-GCCTG | CAGGCSSNNNGGACG |
| T002-GGCAG | CTGCCSSNNNGGACG |
| T003-GGCTG | CAGCCSSNNNGGACG |
| T004-AACGC | GCGTTSSNNNGGACG |
| T005-GCGTT | AACGCSSNNNGGACG |
| T006-ACCGT | ACGGTSSNNNGGACG |
| T007-ACGGT | ACCGTSSNNNGGACG |
| T008-CCGAC | GTCGGSSNNNGGACG |
| T009-CCGTC | GACGGSSNNNGGACG |
| T010-CGACC | GGTCGSSNNNGGACG |
| T011-CGGAC | GTCCGSSNNNGGACG |
| T012-CGGTC | GACCGSSNNNGGACG |
| T013-CGTCC | GGACGSSNNNGGACG |
| T014-GACCG | CGGTCSSNNNGGACG |
| T015-GACGG | CCGTCSSNNNGGACG |
| T016-GGACG | CGTCCSSNNNGGACG |
| T017-GGTCG | CGACCSSNNNGGACG |
| T018-GTCCG | CGGACSSNNNGGACG |
| T019-TGCCT | AGGCASSNNNGGACG |
| T020-AGGCA | TGCCTSSNNNGGACG |
| T021-ATGCG | CGCATSSNNNGGACG |
| T022-CGCAT | ATGCGSSNNNGGACG |
| T023-GAGGC | GCCTCSSNNNGGACG |
| T024-GCCTC | GAGGCSSNNNGGACG |
| T025-GCGAA | TTCGCSSNNNGGACG |
| T026-GGAGC | GCTCCSSNNNGGACG |
| T027-GGCTC | GAGCCSSNNNGGACG |
| T028-TTCGC | GCGAASSNNNGGACG |
| T029-ACCGA | TCGGTSSNNNGGACG |
| T030-ACGGA | TCCGTSSNNNGGACG |
| T031-TCCGT | ACGGASSNNNGGACG |
| T032-TCGGT | ACCGASSNNNGGACG |
| T033-AAGCG | CGCTTSSNNNGGACG |
| T034-CGCTT | AAGCGSSNNNGGACG |
| T035-CCGAG | CTCGGSSNNNGGACG |
| T036-CCTCG | CGAGGSSNNNGGACG |
| T037-CGAGG | CCTCGSSNNNGGACG |
| T038-CGGAG | CTCCGSSNNNGGACG |
| T039-CTCCG | CGGAGSSNNNGGACG |
| T040-CTCGG | CCGAGSSNNNGGACG |
| T041-GGTGG | CCACCSSNNNGGACG |
| T042-CACCC | GGGTGSSNNNGGACG |
| T043-CCACC | GGTGGSSNNNGGACG |
| T044-CCCAC | GTGGGSSNNNGGACG |
| T045-GGGTG | CACCCSSNNNGGACG |
| T046-GTGGG | CCCACSSNNNGGACG |
| T047-ATCGC | GCGATSSNNNGGACG |
| T048-GCGAT | ATCGCSSNNNGGACG |
| T049-AGGCT | AGCCTSSNNNGGACG |
| T050-GCACA | TGTGCSSNNNGGACG |
| T051-GTGCA | TGCACSSNNNGGACG |
| T052-TGCAC | GTGCASSNNNGGACG |
| T053-TGTGC | GCACASSNNNGGACG |
| T054-TCCGA | TCGGASSNNNGGACG |
| T055-TCGGA | TCCGASSNNNGGACG |
| T056-GGCAA | TTGCCSSNNNGGACG |
| T057-TTGGC | GCCAASSNNNGGACG |
| T058-ACCCA | TGGGTSSNNNGGACG |
| T059-TGGGT | ACCCASSNNNGGACG |
| T060-ACACG | CGTGTSSNNNGGACG |
| T061-ACGTG | CACGTSSNNNGGACG |
| T062-CACGT | ACGTGSSNNNGGACG |
| T063-CGTGT | ACACGSSNNNGGACG |
| T064-GACCC | GGGTCSSNNNGGACG |
| T065-GGACC | GGTCCSSNNNGGACG |
| T066-GGGAC | GTCCCSSNNNGGACG |
| T067-GGGTC | GACCCSSNNNGGACG |
| T068-GGTCC | GGACCSSNNNGGACG |
| T069-GTCCC | GGGACSSNNNGGACG |
| T070-CAGGG | CCCTGSSNNNGGACG |
| T071-CCCAG | CTGGGSSNNNGGACG |
| T072-CCCTG | CAGGGSSNNNGGACG |
| T073-CCTGG | CCAGGSSNNNGGACG |
| T074-CTGGG | CCCAGSSNNNGGACG |
| T075-AACCG | CGGTTSSNNNGGACG |
| T076-AACGG | CCGTTSSNNNGGACG |
| T077-CCGTT | AACGGSSNNNGGACG |
| T078-CGGTT | AACCGSSNNNGGACG |
| T079-GCGTA | TACGCSSNNNGGACG |
| T080-TACGC | GCGTASSNNNGGACG |
| T081-CAGCA | TGCTGSSNNNGGACG |
| T082-CTGCA | TGCAGSSNNNGGACG |
| T083-TGCAG | CTGCASSNNNGGACG |
| T084-TGCTG | CAGCASSNNNGGACG |
| T085-ACAGC | GCTGTSSNNNGGACG |
| T086-ACTGC | GCAGTSSNNNGGACG |
| T087-AGCAC | GTGCTSSNNNGGACG |
| T088-AGTGC | GCACTSSNNNGGACG |
| T089-ATGGC | GCCATSSNNNGGACG |
| T090-GCACT | AGTGCSSNNNGGACG |
| T091-GCAGT | ACTGCSSNNNGGACG |
| T092-GCCAT | ATGGCSSNNNGGACG |
| T093-GCTGT | ACAGCSSNNNGGACG |
| T094-GGCAT | ATGCCSSNNNGGACG |
| T095-GTGCT | AGCACSSNNNGGACG |
| T096-TCCCA | TGGGASSNNNGGACG |
| T097-TGGGA | TCCCASSNNNGGACG |
| T098-CACGA | TCGTGSSNNNGGACG |
| T099-CGTGA | TCACGSSNNNGGACG |
| T100-TCACG | CGTGASSNNNGGACG |
| T101-TGACG | CGTCASSNNNGGACG |
| T102-TGTCG | CGACASSNNNGGACG |
| T103-AAGGC | GCCTTSSNNNGGACG |
| T104-CGACA | TGTCGSSNNNGGACG |
| T105-CGTCA | TGACGSSNNNGGACG |
| T106-GCCTT | AAGGCSSNNNGGACG |
| T107-GGCTT | AAGCCSSNNNGGACG |
| T108-TCGTG | CACGASSNNNGGACG |
| T109-ACCCT | AGGGTSSNNNGGACG |
| T110-ACGAC | GTCGTSSNNNGGACG |
| T111-ACGTC | GACGTSSNNNGGACG |
| T112-AGGGT | ACCCTSSNNNGGACG |
| T113-GACGT | ACGTCSSNNNGGACG |
| T114-GTCGT | ACGACSSNNNGGACG |
| T115-CCCTC | GAGGGSSNNNGGACG |
| T116-CCGAA | TTCGGSSNNNGGACG |
| T117-CCTCC | GGAGGSSNNNGGACG |
| T118-CGGAA | TTCCGSSNNNGGACG |
| T119-CTCCC | GGGAGSSNNNGGACG |
| T120-TTCCG | CGGAASSNNNGGACG |
| T121-TTCGG | CCGAASSNNNGGACG |
| T122-GCAGA | TCTGCSSNNNGGACG |
| T123-GCTGA | TCAGCSSNNNGGACG |
| T124-TAGCG | CGCTASSNNNGGACG |
| T125-TGCTC | GAGCASSNNNGGACG |
| T126-CGCTA | TAGCGSSNNNGGACG |
| T127-GAGCA | TGCTCSSNNNGGACG |
| T128-GCTCA | TGAGCSSNNNGGACG |
| T129-TCAGC | GCTGASSNNNGGACG |
| T130-TCTGC | GCAGASSNNNGGACG |
| T131-TGAGC | GCTCASSNNNGGACG |
| T132-AGCAG | CTGCTSSNNNGGACG |
| T133-AGCTG | CAGCTSSNNNGGACG |
| T134-CAGCT | AGCTGSSNNNGGACG |
| T135-CTGCT | AGCAGSSNNNGGACG |
| T136-AGGGA | TCCCTSSNNNGGACG |
| T137-GACGA | TCGTCSSNNNGGACG |
| T138-GTCGA | TCGACSSNNNGGACG |
| T139-TCCCT | AGGGASSNNNGGACG |
| T140-TCGAC | GTCGASSNNNGGACG |
| T141-TCGTC | GACGASSNNNGGACG |
| T142-ACTCG | CGAGTSSNNNGGACG |
| T143-AGACG | CGTCTSSNNNGGACG |
| T144-AGTCG | CGACTSSNNNGGACG |
| T145-ATCCG | CGGATSSNNNGGACG |
| T146-ATCGG | CCGATSSNNNGGACG |
| T147-CCGAT | ATCGGSSNNNGGACG |
| T148-CGAGT | ACTCGSSNNNGGACG |
| T149-CGGAT | ATCCGSSNNNGGACG |
| T150-CTCGT | ACGAGSSNNNGGACG |
| T151-ACGAG | CTCGTSSNNNGGACG |
| T152-CGACT | AGTCGSSNNNGGACG |
| T153-CGTCT | AGACGSSNNNGGACG |
| T154-CACCA | TGGTGSSNNNGGACG |
| T155-CCACA | TGTGGSSNNNGGACG |
| T156-GCAAC | GTTGCSSNNNGGACG |
| T157-GTTGC | GCAACSSNNNGGACG |
| T158-TGGTG | CACCASSNNNGGACG |
| T159-TGTGG | CCACASSNNNGGACG |
| T160-ACACC | GGTGTSSNNNGGACG |
| T161-ACCAC | GTGGTSSNNNGGACG |
| T162-GGTGT | ACACCSSNNNGGACG |
| T163-GTGGT | ACCACSSNNNGGACG |
| T164-CCCAA | TTGGGSSNNNGGACG |
| T165-TTGGG | CCCAASSNNNGGACG |
| T166-AACCC | GGGTTSSNNNGGACG |
| T167-GGGTT | AACCCSSNNNGGACG |
| T168-CAACG | CGTTGSSNNNGGACG |
| T169-CGTTG | CAACGSSNNNGGACG |
| T170-AGAGC | GCTCTSSNNNGGACG |
| T171-AGCTC | GAGCTSSNNNGGACG |
| T172-GAGCT | AGCTCSSNNNGGACG |
| T173-GCTCT | AGAGCSSNNNGGACG |
| T174-TGCAA | TTGCASSNNNGGACG |
| T175-TTGCA | TGCAASSNNNGGACG |
| T176-CATGC | GCATGSSNNNGGACG |
| T177-GCATG | CATGCSSNNNGGACG |
| T178-CGAGA | TCTCGSSNNNGGACG |
| T179-CTCGA | TCGAGSSNNNGGACG |
| T180-TCGAG | CTCGASSNNNGGACG |
| T181-TCTCG | CGAGASSNNNGGACG |
| T182-CCGTA | TACGGSSNNNGGACG |
| T183-CGGTA | TACCGSSNNNGGACG |
| T184-GACCA | TGGTCSSNNNGGACG |
| T185-GGACA | TGTCCSSNNNGGACG |
| T186-GGTCA | TGACCSSNNNGGACG |
| T187-GGTGA | TCACCSSNNNGGACG |
| T188-GTCCA | TGGACSSNNNGGACG |
| T189-GTGGA | TCCACSSNNNGGACG |
| T190-TACCG | CGGTASSNNNGGACG |
| T191-TACGG | CCGTASSNNNGGACG |
| T192-TCACC | GGTGASSNNNGGACG |
| T193-TCCAC | GTGGASSNNNGGACG |
| T194-TGACC | GGTCASSNNNGGACG |
| T195-TGGAC | GTCCASSNNNGGACG |
| T196-TGGTC | GACCASSNNNGGACG |
| T197-TGTCC | GGACASSNNNGGACG |
| T198-CAAGC | GCTTGSSNNNGGACG |
| T199-CTTGC | GCAAGSSNNNGGACG |
| T200-GCAAG | CTTGCSSNNNGGACG |
| T201-GCTTG | CAAGCSSNNNGGACG |
| T202-ACAGG | CCTGTSSNNNGGACG |
| T203-ACCAG | CTGGTSSNNNGGACG |
| T204-ACCTG | CAGGTSSNNNGGACG |
| T205-ACTGG | CCAGTSSNNNGGACG |
| T206-AGGTG | CACCTSSNNNGGACG |
| T207-AGTGG | CCACTSSNNNGGACG |
| T208-ATGGG | CCCATSSNNNGGACG |
| T209-CACCT | AGGTGSSNNNGGACG |
| T210-CAGGT | ACCTGSSNNNGGACG |
| T211-CTGGT | ACCAGSSNNNGGACG |
| T212-AACGT | ACGTTSSNNNGGACG |
| T213-ACGTT | AACGTSSNNNGGACG |
| T214-GGGAA | TTCCCSSNNNGGACG |
| T215-TTCCC | GGGAASSNNNGGACG |
| T216-TGCAT | ATGCASSNNNGGACG |
| T217-ATGCA | TGCATSSNNNGGACG |
| T218-AAGGG | CCCTTSSNNNGGACG |
| T219-CCCTT | AAGGGSSNNNGGACG |
| T220-CGAAC | GTTCGSSNNNGGACG |
| T221-CGTTC | GAACGSSNNNGGACG |
| T222-GAACG | CGTTCSSNNNGGACG |
| T223-GTTCG | CGAACSSNNNGGACG |
| T224-GCCTA | TAGGCSSNNNGGACG |
| T225-GGCTA | TAGCCSSNNNGGACG |
| T226-AAGCA | TGCTTSSNNNGGACG |
| T227-AGCAA | TTGCTSSNNNGGACG |
| T228-GATGC | GCATCSSNNNGGACG |
| T229-GCATC | GATGCSSNNNGGACG |
| T230-TGCTT | AAGCASSNNNGGACG |
| T231-TTGCT | AGCAASSNNNGGACG |
| T232-CAGGA | TCCTGSSNNNGGACG |
| T233-CCAGA | TCTGGSSNNNGGACG |
| T234-CCTCA | TGAGGSSNNNGGACG |
| T235-CCTGA | TCAGGSSNNNGGACG |
| T236-CTCCA | TGGAGSSNNNGGACG |
| T237-CTGGA | TCCAGSSNNNGGACG |
| T238-GAAGC | GCTTCSSNNNGGACG |
| T239-GCTTC | GAAGCSSNNNGGACG |
| T240-TCAGG | CCTGASSNNNGGACG |
| T241-TCCAG | CTGGASSNNNGGACG |
| T242-TCCTG | CAGGASSNNNGGACG |
| T243-TCTGG | CCAGASSNNNGGACG |
| T244-TGAGG | CCTCASSNNNGGACG |
| T245-TGGAG | CTCCASSNNNGGACG |
| T246-AACGA | TCGTTSSNNNGGACG |
| T247-ACCTC | GAGGTSSNNNGGACG |
| T248-ACGAA | TTCGTSSNNNGGACG |
| T249-ACTCC | GGAGTSSNNNGGACG |
| T250-AGACC | GGTCTSSNNNGGACG |
| T251-AGGAC | GTCCTSSNNNGGACG |
| T252-AGTCC | GGACTSSNNNGGACG |
| T253-ATCCC | GGGATSSNNNGGACG |
| T254-GACCT | AGGTCSSNNNGGACG |
| T255-GAGGT | ACCTCSSNNNGGACG |
| T256-GGACT | AGTCCSSNNNGGACG |
| T257-GGAGT | ACTCCSSNNNGGACG |
| T258-GGGAT | ATCCCSSNNNGGACG |
| T259-GGTCT | AGACCSSNNNGGACG |
| T260-GTCCT | AGGACSSNNNGGACG |
| T261-TCGTT | AACGASSNNNGGACG |
| T262-TTCGT | ACGAASSNNNGGACG |
| T263-AGGTC | GACCTSSNNNGGACG |
| T264-CATCG | CGATGSSNNNGGACG |
| T265-CGATG | CATCGSSNNNGGACG |
| T266-CACAC | GTGTGSSNNNGGACG |
| T267-GTGTG | CACACSSNNNGGACG |
| T268-AGCAT | ATGCTSSNNNGGACG |
| T269-CGAAG | CTTCGSSNNNGGACG |
| T270-CTTCG | CGAAGSSNNNGGACG |
| T271-ATGCT | AGCATSSNNNGGACG |
| T272-CAACC | GGTTGSSNNNGGACG |
| T273-CCAAC | GTTGGSSNNNGGACG |
| T274-AAGCT | AGCTTSSNNNGGACG |
| T275-ACGAT | ATCGTSSNNNGGACG |
| T276-ATCGT | ACGATSSNNNGGACG |
| T277-ACACA | TGTGTSSNNNGGACG |
| T278-TGTGT | ACACASSNNNGGACG |
| T279-TCCTC | GAGGASSNNNGGACG |
| T280-TCGAA | TTCGASSNNNGGACG |
| T281-AGAGG | CCTCTSSNNNGGACG |
| T282-AGGAG | CTCCTSSNNNGGACG |
| T283-CCTCT | AGAGGSSNNNGGACG |
| T284-CGATC | GATCGSSNNNGGACG |
| T285-CTCCT | AGGAGSSNNNGGACG |
| T286-GATCG | CGATCSSNNNGGACG |
| T287-GGGTA | TACCCSSNNNGGACG |
| T288-TACCC | GGGTASSNNNGGACG |
| T289-GCAAA | TTTGCSSNNNGGACG |
| T290-AACCA | TGGTTSSNNNGGACG |
| T291-ACCAA | TTGGTSSNNNGGACG |
| T292-CGTAC | GTACGSSNNNGGACG |
| T293-GACAC | GTGTCSSNNNGGACG |
| T294-GTACG | CGTACSSNNNGGACG |
| T295-GTCAC | GTGACSSNNNGGACG |
| T296-GTGAC | GTCACSSNNNGGACG |
| T297-GTGTC | GACACSSNNNGGACG |
| T298-CACAG | CTGTGSSNNNGGACG |
| T299-CACTG | CAGTGSSNNNGGACG |
| T300-CAGTG | CACTGSSNNNGGACG |
| T301-CATGG | CCATGSSNNNGGACG |
| T302-CCATG | CATGGSSNNNGGACG |
| T303-CTGTG | CACAGSSNNNGGACG |
| T304-GAACC | GGTTCSSNNNGGACG |
| T305-GGAAC | GTTCCSSNNNGGACG |
| T306-GGTTC | GAACCSSNNNGGACG |
| T307-GTTCC | GGAACSSNNNGGACG |
| T308-CAAGG | CCTTGSSNNNGGACG |
| T309-CCAAG | CTTGGSSNNNGGACG |
| T310-CCTTG | CAAGGSSNNNGGACG |
| T311-CTTGG | CCAAGSSNNNGGACG |
| T312-AAACG | CGTTTSSNNNGGACG |
| T313-TCGAT | ATCGASSNNNGGACG |
| T314-ATCGA | TCGATSSNNNGGACG |
| T315-GCTAC | GTAGCSSNNNGGACG |
| T316-GTAGC | GCTACSSNNNGGACG |
| T317-TCACA | TGTGASSNNNGGACG |
| T318-TGACA | TGTCASSNNNGGACG |
| T319-TGTCA | TGACASSNNNGGACG |
| T320-TGTGA | TCACASSNNNGGACG |
| T321-ACGTA | TACGTSSNNNGGACG |
| T322-TACGT | ACGTASSNNNGGACG |
| T323-GCAAT | ATTGCSSNNNGGACG |
| T324-GCATT | AATGCSSNNNGGACG |
| T325-AATGC | GCATTSSNNNGGACG |
| T326-ATTGC | GCAATSSNNNGGACG |
| T327-ACAGT | ACTGTSSNNNGGACG |
| T328-ACCAT | ATGGTSSNNNGGACG |
| T329-ACTGT | ACAGTSSNNNGGACG |
| T330-AGTGT | ACACTSSNNNGGACG |
| T331-ATGGT | ACCATSSNNNGGACG |
| T332-ACACT | AGTGTSSNNNGGACG |
| T333-TCCAA | TTGGASSNNNGGACG |
| T334-TGGAA | TTCCASSNNNGGACG |
| T335-TTCCA | TGGAASSNNNGGACG |
| T336-TTGGA | TCCAASSNNNGGACG |
| T337-AAAGC | GCTTTSSNNNGGACG |
| T338-CACTC | GAGTGSSNNNGGACG |
| T339-CAGAC | GTCTGSSNNNGGACG |
| T340-CAGTC | GACTGSSNNNGGACG |
| T341-CATCC | GGATGSSNNNGGACG |
| T342-CCATC | GATGGSSNNNGGACG |
| T343-CGTAG | CTACGSSNNNGGACG |
| T344-CTACG | CGTAGSSNNNGGACG |
| T345-CTCAC | GTGAGSSNNNGGACG |
| T346-CTGAC | GTCAGSSNNNGGACG |
| T347-CTGTC | GACAGSSNNNGGACG |
| T348-GACAG | CTGTCSSNNNGGACG |
| T349-GACTG | CAGTCSSNNNGGACG |
| T350-GAGTG | CACTCSSNNNGGACG |
| T351-GATGG | CCATCSSNNNGGACG |
| T352-GGATG | CATCCSSNNNGGACG |
| T353-GTCAG | CTGACSSNNNGGACG |
| T354-GTCTG | CAGACSSNNNGGACG |
| T355-GTGAG | CTCACSSNNNGGACG |
| T356-AACCT | AGGTTSSNNNGGACG |
| T357-AAGGT | ACCTTSSNNNGGACG |
| T358-ACCTT | AAGGTSSNNNGGACG |
| T359-AGGTT | AACCTSSNNNGGACG |
| T360-TAGCA | TGCTASSNNNGGACG |
| T361-TGCTA | TAGCASSNNNGGACG |
| T362-CCTTC | GAAGGSSNNNGGACG |
| T363-CGAAA | TTTCGSSNNNGGACG |
| T364-CTTCC | GGAAGSSNNNGGACG |
| T365-GAAGG | CCTTCSSNNNGGACG |
| T366-GGAAG | CTTCCSSNNNGGACG |
| T367-TACGA | TCGTASSNNNGGACG |
| T368-TCGTA | TACGASSNNNGGACG |
| T369-CTAGC | GCTAGSSNNNGGACG |
| T370-GCTAG | CTAGCSSNNNGGACG |
| T371-ACAGA | TCTGTSSNNNGGACG |
| T372-ACTCA | TGAGTSSNNNGGACG |
| T373-ACTGA | TCAGTSSNNNGGACG |
| T374-AGACA | TGTCTSSNNNGGACG |
| T375-AGTCA | TGACTSSNNNGGACG |
| T376-AGTGA | TCACTSSNNNGGACG |
| T377-ATCCA | TGGATSSNNNGGACG |
| T378-ATGGA | TCCATSSNNNGGACG |
| T379-TCCAT | ATGGASSNNNGGACG |
| T380-TGACT | AGTCASSNNNGGACG |
| T381-TGTCT | AGACASSNNNGGACG |
| T382-GACTC | GAGTCSSNNNGGACG |
| T383-GAGTC | GACTCSSNNNGGACG |
| T384-GATCC | GGATCSSNNNGGACG |
| T385-GGATC | GATCCSSNNNGGACG |
| T386-GTCTC | GAGACSSNNNGGACG |
| T387-CTCTG | CAGAGSSNNNGGACG |
| T388-CAGAG | CTCTGSSNNNGGACG |
| T389-CTCAG | CTGAGSSNNNGGACG |
| T390-CTGAG | CTCAGSSNNNGGACG |
| T391-AATCG | CGATTSSNNNGGACG |
| T392-ATTCG | CGAATSSNNNGGACG |
| T393-CGAAT | ATTCGSSNNNGGACG |
| T394-CGATT | AATCGSSNNNGGACG |
| T395-GGTAC | GTACCSSNNNGGACG |
| T396-GTACC | GGTACSSNNNGGACG |
| T397-CAACA | TGTTGSSNNNGGACG |
| T398-CACAA | TTGTGSSNNNGGACG |
| T399-TGTTG | CAACASSNNNGGACG |
| T400-TTGTG | CACAASSNNNGGACG |
| T401-AACAC | GTGTTSSNNNGGACG |
| T402-ACAAC | GTTGTSSNNNGGACG |
| T403-AGCTA | TAGCTSSNNNGGACG |
| T404-GTGTT | AACACSSNNNGGACG |
| T405-GTTGT | ACAACSSNNNGGACG |
| T406-TAGCT | AGCTASSNNNGGACG |
| T407-CCAAA | TTTGGSSNNNGGACG |
| T408-TCAGA | TCTGASSNNNGGACG |
| T409-TCTCA | TGAGASSNNNGGACG |
| T410-TCTGA | TCAGASSNNNGGACG |
| T411-TGAGA | TCTCASSNNNGGACG |
| T412-TACCA | TGGTASSNNNGGACG |
| T413-TGGTA | TACCASSNNNGGACG |
| T414-ACTCT | AGAGTSSNNNGGACG |
| T415-AGACT | AGTCTSSNNNGGACG |
| T416-AGAGT | ACTCTSSNNNGGACG |
| T417-AGGAT | ATCCTSSNNNGGACG |
| T418-AGTCT | AGACTSSNNNGGACG |
| T419-ATCCT | AGGATSSNNNGGACG |
| T420-ACATG | CATGTSSNNNGGACG |
| T421-ATGTG | CACATSSNNNGGACG |
| T422-CACAT | ATGTGSSNNNGGACG |
| T423-CATGT | ACATGSSNNNGGACG |
| T424-CTCTC | GAGAGSSNNNGGACG |
| T425-GAGAG | CTCTCSSNNNGGACG |
| T426-CCTAC | GTAGGSSNNNGGACG |
| T427-CGTAA | TTACGSSNNNGGACG |
| T428-CGTTA | TAACGSSNNNGGACG |
| T429-CTACC | GGTAGSSNNNGGACG |
| T430-GAACA | TGTTCSSNNNGGACG |
| T431-GACAA | TTGTCSSNNNGGACG |
| T432-GGTAG | CTACCSSNNNGGACG |
| T433-GTAGG | CCTACSSNNNGGACG |
| T434-GTCAA | TTGACSSNNNGGACG |
| T435-GTGAA | TTCACSSNNNGGACG |
| T436-GTTCA | TGAACSSNNNGGACG |
| T437-GTTGA | TCAACSSNNNGGACG |
| T438-TAACG | CGTTASSNNNGGACG |
| T439-TCAAC | GTTGASSNNNGGACG |
| T440-TGAAC | GTTCASSNNNGGACG |
| T441-TGTTC | GAACASSNNNGGACG |
| T442-TTACG | CGTAASSNNNGGACG |
| T443-TTCAC | GTGAASSNNNGGACG |
| T444-TTGAC | GTCAASSNNNGGACG |
| T445-TTGTC | GACAASSNNNGGACG |
| T446-AACAG | CTGTTSSNNNGGACG |
| T447-AACTG | CAGTTSSNNNGGACG |
| T448-AAGTG | CACTTSSNNNGGACG |
| T449-AATGG | CCATTSSNNNGGACG |
| T450-ACAAG | CTTGTSSNNNGGACG |
| T451-ACTTG | CAAGTSSNNNGGACG |
| T452-AGTTG | CAACTSSNNNGGACG |
| T453-ATTGG | CCAATSSNNNGGACG |
| T454-CAACT | AGTTGSSNNNGGACG |
| T455-CAAGT | ACTTGSSNNNGGACG |
| T456-CCAAT | ATTGGSSNNNGGACG |
| T457-CCATT | AATGGSSNNNGGACG |
| T458-CTGTT | AACAGSSNNNGGACG |
| T459-GCATA | TATGCSSNNNGGACG |
| T460-TATGC | GCATASSNNNGGACG |
| T461-GGAAA | TTTCCSSNNNGGACG |
| T462-AGAGA | TCTCTSSNNNGGACG |
| T463-TCTCT | AGAGASSNNNGGACG |
| T464-GCTAA | TTAGCSSNNNGGACG |
| T465-GCTTA | TAAGCSSNNNGGACG |
| T466-TAAGC | GCTTASSNNNGGACG |
| T467-TTAGC | GCTAASSNNNGGACG |
| T468-ACCTA | TAGGTSSNNNGGACG |
| T469-AGGTA | TACCTSSNNNGGACG |
| T470-TACCT | AGGTASSNNNGGACG |
| T471-TAGGT | ACCTASSNNNGGACG |
| T472-CATCA | TGATGSSNNNGGACG |
| T473-CATGA | TCATGSSNNNGGACG |
| T474-TGATG | CATCASSNNNGGACG |
| T475-ATACG | CGTATSSNNNGGACG |
| T476-ACATC | GATGTSSNNNGGACG |
| T477-ATCAC | GTGATSSNNNGGACG |
| T478-ATGAC | GTCATSSNNNGGACG |
| T479-ATGTC | GACATSSNNNGGACG |
| T480-CAAGA | TCTTGSSNNNGGACG |
| T481-CAGAA | TTCTGSSNNNGGACG |
| T482-CCTAG | CTAGGSSNNNGGACG |
| T483-CTAGG | CCTAGSSNNNGGACG |
| T484-CTCAA | TTGAGSSNNNGGACG |
| T485-CTGAA | TTCAGSSNNNGGACG |
| T486-CTTCA | TGAAGSSNNNGGACG |
| T487-CTTGA | TCAAGSSNNNGGACG |
| T488-TCAAG | CTTGASSNNNGGACG |
| T489-TCTTG | CAAGASSNNNGGACG |
| T490-TGAAG | CTTCASSNNNGGACG |
| T491-TTCAG | CTGAASSNNNGGACG |
| T492-TTCTG | CAGAASSNNNGGACG |
| T493-TTGAG | CTCAASSNNNGGACG |
| T494-AACTC | GAGTTSSNNNGGACG |
| T495-AAGAC | GTCTTSSNNNGGACG |
| T496-AAGTC | GACTTSSNNNGGACG |
| T497-ACTTC | GAAGTSSNNNGGACG |
| T498-AGAAC | GTTCTSSNNNGGACG |
| T499-AGTTC | GAACTSSNNNGGACG |
| T500-ATTCC | GGAATSSNNNGGACG |
| T501-ATAGC | GCTATSSNNNGGACG |
| T502-TAGGA | TCCTASSNNNGGACG |
| T503-TCCTA | TAGGASSNNNGGACG |
| T504-CGATA | TATCGSSNNNGGACG |
| T505-GATCA | TGATCSSNNNGGACG |
| T506-GATGA | TCATCSSNNNGGACG |
| T507-AGATG | CATCTSSNNNGGACG |
| T508-ATCAG | CTGATSSNNNGGACG |
| T509-ATCTG | CAGATSSNNNGGACG |
| T510-ATGAG | CTCATSSNNNGGACG |
| T511-GTACA | TGTACSSNNNGGACG |
| T512-GTGTA | TACACSSNNNGGACG |
